# Efficacy of Molnupiravir for the Treatment of Mild or Moderate COVID-19 in Adults: A Meta-Analysis

**DOI:** 10.7759/cureus.38586

**Published:** 2023-05-05

**Authors:** Karima Benaicha, Raja Ram Khenhrani, Maha Veer, Sapna Devi, Usman Shahbaz, Qais M Salah, Mostafa Hammad, Sujith K Palleti

**Affiliations:** 1 Internal Medicine, University Hospital Isaad Hassani, Algiers, DZA; 2 Internal Medicine, Liaquat University of Medical and Health Sciences, Karachi, PAK; 3 Medicine, Liaquat University of Medical and Health Sciences, Karachi, PAK; 4 Medical School, Liaquat University of Medical and Health Sciences, Karachi, PAK; 5 Medicine, Allama Iqbal Medical College, Lahore, PAK; 6 Internal Medicine, Al Quds University Faculty of Medicine, Jerusalem, PSE; 7 Medicine, New Giza University, New Giza, EGY; 8 Nephrology, Edward Hines Jr. Veterans Administration Hospital, Hines, USA; 9 Nephrology, Loyola University Medical Center, Maywood, USA

**Keywords:** meta-analysis, efficacy, adults, covid-19, molnupiravir

## Abstract

The aim of this meta-analysis is to evaluate the efficacy of molnupiravir among mild or moderate COVID-19 patients. This meta-analysis was reported according to the guidelines of Preferred Reporting Items for Systematic Reviews and Meta-Analyses. Two authors independently performed a comprehensive search for relevant studies in PubMed, Cochrane Library, and Web of Science. The keywords used to search for relevant records were “Molnupiravir,” “COVID-19,” and “efficacy.” This meta-analysis included studies that compared the effectiveness of molnupiravir with a placebo for COVID-19 treatment. The primary outcome assessed in this meta-analysis was the composite of hospitalization and all-cause mortality (30 days). In addition, we assessed all-cause mortality and hospitalization separately and the number of patients who tested negative for viral RNA on day five. A total of 10 studies were included in the meta-analysis. Among the 10 studies, five were randomized controlled trials and five were observational studies. Based on the results presented in the meta-analysis, it can be concluded that molnupiravir has a significant impact on reducing all-cause mortality and improving the proportion of patients who test negative for viral RNA on day five. The risk of hospitalization and composite outcome was also lower in molnupiravir-treated patients, although the difference was statistically insignificant. The subgroup analysis showed consistent results across all subgroups, indicating that the effect of molnupiravir is consistent regardless of patient characteristics.

## Introduction and background

The COVID-19 pandemic, caused by SARS-CoV-2, has caused a significant rise in morbidity and mortality worldwide. The rapid spread of the disease and the increasing number of infections led the World Health Organization (WHO) to declare it a pandemic in 2020 [1]. The clinical profile of COVID-19 ranges from symptomatic events to life-threatening events, with a high number of patients dying from shock, respiratory failure, and multiple organ failure [2]. The genetic variability of viruses presents an additional challenge because subsequent variants are dissimilar not only in their pathogenicity and infectivity but also in the efficiency of the drugs used to fight against them [2]. Although vaccination may be the most effective way to control COVID-19, it will take a long time for the public to widely accept and participate in vaccination programs, especially in underdeveloped regions. Moreover, there have been increasing reports of vaccinated individuals contracting SARS-CoV-2 infection globally. Hence, there is an urgent need for oral antiviral therapy as it can effectively prevent the progression of the disease and halt the transmission of the virus.

A few antiviral drugs have been proven to decrease the risk of progression to severe illness in high-risk patients [3,4]. One of these drugs is molnupiravir. It is an antiviral drug that can be taken orally and works by inhibiting RdRp. It is a prodrug of β-D-N4-hydroxycytidine, a ribonucleoside analog that can reduce the viral load and inhibit the replication of SARS-CoV-2. A significant advantage of molnupiravir is its favorable pharmacokinetic profile, which means it can be used by a wide range of people [5].

Molnupiravir is an effective treatment for coronaviruses, including different variants of SARS-CoV-2, and has a low risk of drug resistance development [6]. The recommended dosage for adults over 18 years old is 800 mg, taken orally every 12 hours for five days. Molnupiravir should be started within five days of symptom onset in COVID-19 patients. Due to its prodrug nature, it is metabolized by human esterases into its active form, making the likelihood of drug interactions limited. Molnupiravir is easy to administer at home and does not interact with other chronic treatments [7]. The Food and Drug Administration (FDA) approved this drug for emergency use in the treatment of mild-to-moderate COVID-19 infection after phase II and phase III of the MOVE-OUT trial in non-hospitalized patients with COVID-19 infection [8]. Several other studies have shown that the SARS-CoV-2 Omicron variant demonstrates high transmissibility, partial vaccine escape, and lesser clinical severity of disease compared to the Delta variant [9,10]. A previously conducted meta-analysis showed the beneficial impact of molnupiravir in patients with mild or moderate COVID-19 [11]. Since then, new studies have been conducted. Therefore, the present meta-analysis has been conducted to evaluate the efficacy of molnupiravir among mild or moderate COVID-19 patients.

## Review

Methodology

The present meta-analysis was reported according to the guidelines of Preferred Reporting Items for Systematic Reviews and Meta-Analyses (PRISMA).

Data Search and Eligibility Criteria

Two authors independently performed a comprehensive search for relevant studies in PubMed, Cochrane Library, and Web of Science. The keywords used to search for relevant records were “Molnupiravir,” “COVID-19,” and “efficacy.” The present meta-analysis included studies that compared the effectiveness of molnupiravir with placebo or usual care in COVID-19 treatment. We included all prospective and retrospective studies but excluded studies published in languages other than English. Reviews, case reports, case series, and editorials were also excluded. Studies that did not report desired outcomes were not included in the present meta-analysis. All eligible studies were imported into ENDNOTE X9, and after removing duplicates, initial screening was done using titles and abstracts. The full text of eligible records was retrieved, and a detailed assessment of eligibility criteria was done by two authors independently. Any disagreements in the process of study search and selection were resolved via consensus.

Data Extraction and Outcome Measures

Data from the included studies were manually extracted using a pre-designed Microsoft Excel spreadsheet. The data extracted from the studies included author name, year of publication, study design, region, dosage of molnupiravir, sample size, and outcome measures. One author extracted the data, and the second cross-checked the data from the studies and entered it into RevMan for analysis. The primary outcome assessed in this meta-analysis was the composite of hospitalization and all-cause mortality. We also assessed all-cause mortality (30 days) and hospitalization separately and the number of patients who tested negative for viral RNA on day five. The quality of included studies was assessed by two authors independently using the Cochrane Risk of Bias Assessment Tool for randomized controlled trials (RCTs) and the Newcastle-Ottawa Scale (NOS) for observational studies.

Data Analysis

Data analysis was performed using RevMan Version 5.4.1. For binary outcomes, risk ratios (RRs) and 95% confidence intervals (CIs) were calculated, and a p-value of less than 0.05 was considered significant. The degree of statistical heterogeneity was assessed using the I^2^ test, and the Q-statistic test was used to define the level of heterogeneity. When the p-value was less than 0.10 for the Q-test and I^2^ was more than 50%, studies were considered significant for heterogeneity. The fixed-effects model was applied when I^2^ was less than 50%, and the random-effect model was used otherwise. Subgroup analysis was conducted to determine the impact of molnupiravir on the primary outcome in different groups, including males, females, diabetics, non-diabetics, and vaccinated and non-vaccinated patients.

Results

**Figure 1:**
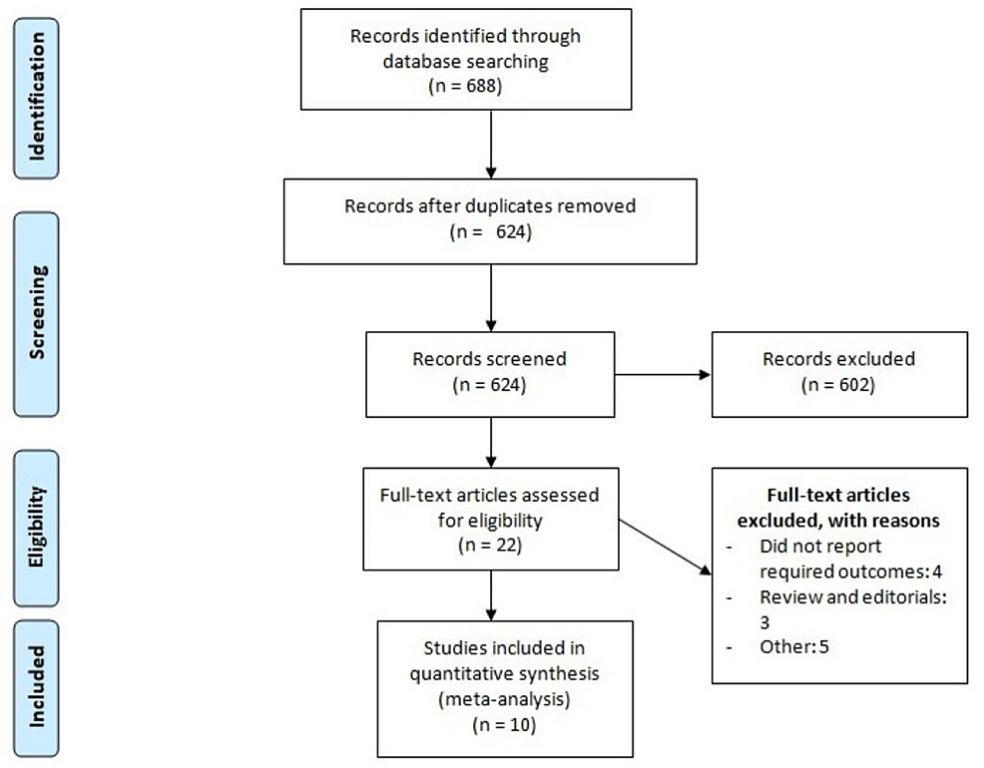
Process of study selection.

**Figure 2:**
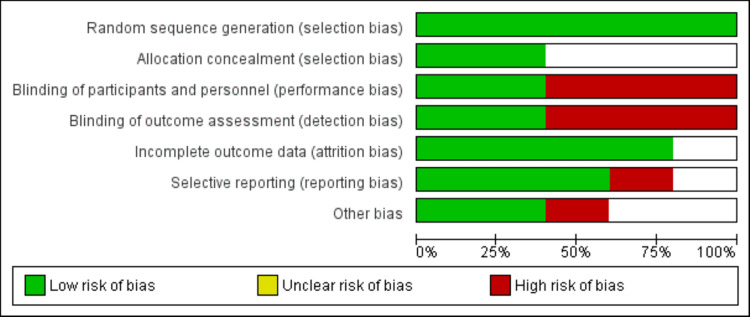
Quality assessment of RCTs. RCT: randomized controlled trial

Figure 1 shows the PRISMA flowchart of the study selection process. Initially, 688 studies were identified through database searching. After removing duplicates, 624 articles were screened based on their titles and abstracts. The full text of 22 articles was obtained, and a detailed assessment was conducted based on pre-defined inclusion and exclusion criteria. Finally, 10 studies were included in the meta-analysis. Table 1 presents the characteristics of the included studies. Among the 10 studies, five were RCTs and five were observational studies. Figure 2 shows the quality assessment of included RCTs. Out of five RCTs, only two were double-blinded and the remaining three were open-label. Table 2 shows the quality assessment of observational studies.

**Table 1:** Characteristics of included studies. RCT: randomized control trial; NR: not reported; BID: two times a day

Author name	Publication year	Study design	Groups	Dose	Sample size	Age (years)	Male (%)
Bernal et al. [8]	2022	RCT	Molnupiravir	800 mg BID	709	43 vs. 44	48.7 vs. 51
			Placebo		699		
Butler et al. [12]	2023	RCT	Molnupiravir	800 mg BID	12,529	56.7 vs. 56.5	42 vs. 21
			Placebo		12,525		
Fischer et al. [13]	2021	RCT	Molnupiravir	200, 400, or 800 mg	117	NR	40.2 vs. 45.2
			Placebo		54		
Flisiak et al. [2]	2022	Observational	Molnupiravir	800 mg BID	96	67.4 vs. 67.4	44 vs. 45
			Placebo		159		
Najjar-Debbiny et al. [14]	2023	Observational	Molnupiravir	NR	2,661	73.1 vs. 73.1	50.4 vs. 49.8
			Placebo		2,661		
Sinha et al. [15]	2022	RCT	Molnupiravir	800 mg BID	608	NR	67.1 vs. 69.7
			Placebo		610		
Suzuki et al. [16]	2022	Observational	Molnupiravir	NR	230	64.1 vs. 64.7	53 vs. 51.2
			Placebo		690		
Wong et al. [17]	2022	Observational	Molnupiravir	800 mg BID	4,983	NR	47.5 vs. 47.8
			Placebo		49,234		
Xie et al. [18]	2023	Observational	Molnupiravir	NR	7,818	69.2 vs. 67.1	91.2 vs. 89.4
			Placebo		78,180		
Zou et al. [19]	2022	RCT	Molnupiravir	800 mg BID	77	39 vs. 42	55.8 vs. 54.8
			Placebo		31		

**Table 2:** Quality assessment of observational studies.

Study	Selection	Comparability	Outcome	Overall
Flisiak et al. [2]	3	2	2	Good
Najjar-Debbiny et al. [14]	3	2	1	Fair
Suzuki et al. [16]	3	1	2	Fair
Wong et al. [17]	2	2	2	Fair
Xie et al. [18]	4	2	2	Good

All-Cause Mortality and Hospitalization

**Figure 3:**
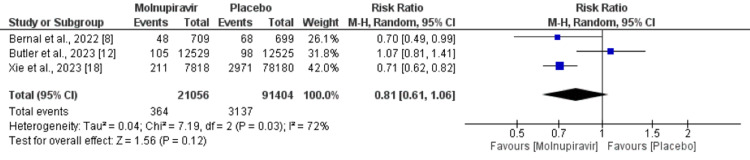
Effect of molnupiravir on the composite outcome. Sources: References [8,12,18].

Three studies involving 112,460 patients (21,056 received molnupiravir and 91,404 received placebo) reported the composite of all-cause mortality and hospitalization. There was no significant difference between molnupiravir-treated and placebo-treated patients in terms of composite outcome (RR = 0.81, 95% CI = 0.61-1.06), as shown in Figure 3. Significant heterogeneity was reported among the study results (I^2^ = 72%). We performed a sensitivity analysis by removing the study conducted by Butler et al. After removing this study, we found the risk of composite all-cause mortality and hospitalization to be higher in placebo-treated patients compared to molnupiravir-treated patients (RR = 0.71, 95% CI = 0.62-0.81). Heterogeneity was reduced to 0% from 72%.

**Figure 4:**
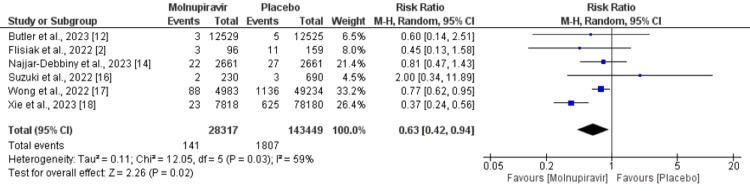
Effect of molnupiravir on all-cause mortality. Sources: References [2,12,14,16-18].

**Figure 5:**
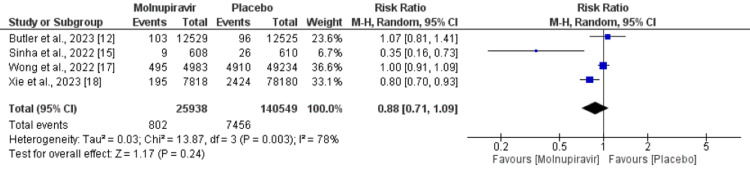
Effect of molnupiravir on hospitalization. Sources: References [12,15,17,18].

We analyzed all-cause mortality and hospitalization separately. A total of six studies were included in the pooled analysis of all-cause mortality including a total of 171,766 patients. All-cause mortality was significantly higher in patients receiving placebo therapy compared to molnupiravir-treated patients (RR = 0.63, 95% CI = 0.42-0.94), as shown in Figure 4. Significant heterogeneity was reported among the study results (I^2^ = 59%). Four studies reported hospitalization including 166,487 patients. The hospitalization rate was higher in patients who received a placebo (5.30%) compared to molnupiravir-treated patients (2.32%). However, the difference was statistically insignificant (RR = 0.93, 95% CI = 0.87-1.00), as shown in Figure 5. Significant heterogeneity was reported among the study results (I^2^ = 78%).

Number of Patients Testing Negative for Viral RNA on Day Five

Three studies compared the proportion of patients who tested negative for viral RNA on day five. The number of patients who were negative for viral RNA on day five was significantly greater in patients receiving molnupiravir compared to placebo (RR = 5.18, 95% CI = 1.64-16.37). Significant heterogeneity was reported among the study results (I^2^ = 83%).

Subgroup Analysis

We performed a subgroup analysis to determine the impact of molnupiravir on different groups of people including males, females, patients with diabetes and without diabetes, and vaccinated and non-vaccinated patients. The results of the subgroup analysis are shown in Table 3. We found no significant difference between vaccinated and non-vaccinated individuals, males and females, and patients with diabetes and without diabetes as the effect of molnupiravir was consistent across all subgroups.

**Table 3:** Results of the subgroup analysis. *: Significant at p-value <0.05. RR: risk ratio; CI: confidence interval; NA: not applicable

Groups	Number of studies	RR (95% CI)	I-square	P-value for subgroup difference
Males	3	0.75 (0.68-0.83)*	0%	0.07
Females	3	0.61 (0.50-0.75)*	0%	
Diabetes (yes)	3	0.72 (0.62-0.84)*	47%	0.46
Diabetes (no)	3	0.77 (0.72-0.81)*	32%	
COVID-19 vaccination (yes)	1	0.69 (0.57, 0.83)*	NA	0.13
COVID-19 vaccination (no)	1	0.83 (0.71, 0.97)*	NA	

Discussion

This meta-analysis included 10 studies that assessed the benefits of molnupiravir in adult patients with COVID-19 and found a significant impact of molnupiravir on all-cause mortality and the proportion of patients who tested negative for viral RNA on day five. The meta-analysis reported that the risk of all-cause mortality was significantly lower in the molnupiravir group compared to placebo. Additionally, the number of patients who tested negative for viral RNA on day five was significantly higher in the molnupiravir group. However, the risk of hospitalization was lower in molnupiravir-treated patients, but the difference was statistically insignificant (p = 0.24). A meta-analysis conducted by Huang et al. [11] included six studies and reported that the risk of death was decreased by 34%, and the risk of the composite outcome of disease progression was decreased by 37% among patients who received molnupiravir.

A study conducted by Arribas et al. [20] did not report any clinical benefit of molnupiravir therapy in the treatment of COVID-19. Per the COVID-19 treatment guidelines from the National Institutes of Health, adult patients who are at a high risk of disease progression and those who have not been hospitalized should be given molnupiravir orally twice a day for five days at a dose of 800 mg. It is recommended to start the treatment as early as possible within five days of the initial signs or symptoms [21]. In the study conducted by Arribas et al., the majority of patients had severe COVID-19, and the molnupiravir treatment was started more than five days after the onset of symptoms [20]. Thus, this study did not demonstrate any benefits of molnupiravir.

Although the Omicron variant of COVID-19 has shown reduced responsiveness to vaccines and certain COVID-19 treatments, laboratory studies suggest that antiviral medications such as remdesivir, nirmatrelvir/ritonavir, and molnupiravir continue to be effective against this particular strain [22]. This meta-analysis reported a high proportion of patients who tested negative for viral RNA on day five. Additionally, Butler et al. [12] assessed the days to self-reported recovery. The days to self-reported recovery were significantly lower in patients receiving molnupiravir. Sinha et al. [15] reported that the proportion of patients with clinical improvement from baseline at day five was significantly higher in molnupiravir-treated patients compared to placebo (80.8% vs. 32.1%). Multiple studies included in this meta-analysis reported that molnupiravir can decrease viral loads quicker compared to a placebo. This specific benefit of faster reduction in viral loads of patients with COVID-19 can reduce the transmission of SARS-CoV-2. It clearly shows the beneficial impact of molnupiravir in COVID-19 patients.

Regarding the rate of hospitalization, four studies assessed this outcome. Pooled analysis showed that the risk of hospitalization was higher in patients receiving molnupiravir (3.1%) compared to placebo (5.3%). However, the difference was statistically insignificant. Out of the four studies included, three studies reported low hospitalization in the molnupiravir group. More RCTs are needed to assess the benefits of molnupiravir related to the rate of hospitalization of non-hospitalized COVID-19 patients.

A recent study conducted by Kamal et al. analyzed previous studies on molnupiravir as a potential treatment for mild-to-moderate COVID-19 infections in high-risk patients. The analysis concluded that both preclinical and clinical studies have shown the effectiveness and safety of molnupiravir as an antiviral therapy [23]. However, most RCTs were done on non-vaccinated participants. Therefore, there is a lack of data on the use of molnupiravir in vaccinated patients. This is an important gap in knowledge as a significant portion of the global population is currently vaccinated, and there is no information about the effectiveness of molnupiravir in treating breakthrough infections following vaccination [1].

The present meta-analysis has certain limitations. First, the results of observational studies were used in the current meta-analysis. Second, studies included in this meta-analysis showed heterogeneity because of different populations, variants, and distinct vaccine histories. In the future, large randomized trials are required that can assess the impact of molnupiravir on different subgroups.

## Conclusions

Based on the results presented in the meta-analysis, it can be concluded that molnupiravir has a significant impact on reducing all-cause mortality and improving the proportion of patients who test negative for viral RNA on day five. The risk of hospitalization was also lower in molnupiravir-treated patients, although the difference was statistically insignificant. The subgroup analysis showed consistent results across all subgroups, indicating that the effect of molnupiravir is consistent regardless of patient characteristics. Overall, these findings suggest that molnupiravir can be an effective treatment option for adult patients with COVID-19. Further studies are needed to explore the efficacy of molnupiravir in treating COVID-19 caused by new variants and in vaccinated and non-vaccinated individuals.
